# Comparison of robotic-assisted and manual cochlear implant electrode array insertions considering cochlear geometric factors

**DOI:** 10.1007/s00405-026-10613-1

**Published:** 2026-10-03

**Authors:** Anni Andersson, Stephan Geiger, Matti Pekka Iso-Mustajärvi, Petro Julkunen, Tim Nauwelaers, Aarno Dietz

**Affiliations:** 1 Department of Otorhinolaryngology, Kuopio University Hospital, Kuopio, Finland; 2 Advanced Bionics LLC, Valencia, CA USA; 3 Department of Technical Physics, University of Eastern Finland, Kuopio, Finland; 4 Institute of Clinical Medicine, University of Eastern Finland, Kuopio, Finland; 5 Department of Clinical Neurophysiology, Kuopio University Hospital, Kuopio, Finland; 6 Advanced Bionics GmbH, European Research Center, Hannover, Germany

**Keywords:** Cochlear implants, Trauma, Robotic-assisted surgery, Cochlear geometry

## Abstract

**Purpose:**

Purpose of the present study was to compare the intracochlear trauma coincidentally caused by robotic-assisted and manual cochlear implant electrode array insertions in human temporal bones, and to evaluate the influence of cochlear geometry on insertion outcomes.

**Materials and methods:**

Twenty human temporal bones, paired according to cochlear size, underwent round window insertion with a HiFocus SlimJ array, either manually or using the iotaSOFT® insertion system. Postoperative cone beam computed tomography (CBCTs) and micro-CTs were used to measure angular insertion depth (AID) and cochlear dimensions, and to assess electrode scalar location to define the level of trauma. Additionally, a cochlea implant imaging tool was used to measure lateral scala tympani height and estimate angular insertion depth.

**Results:**

No scalar translocation occurred in the robotic group, whereas three translocations were found in the manual group. Mean trauma scores were lower for robotic insertions (0.30 ± 0.48) compared with manual insertions (0.89 ± 0.93). Manual insertion produced significantly deeper AID (428 ± 82°) than robotic insertions (304 ± 47°, p = 0.004). The robotic group exhibited significantly more extra-cochlear contacts (2.3 ± 1.8) than the manual group (0.3 ± 0.7, p = 0.011). Greater AID correlated with more severe trauma (r = 0.682, p = 0.001). Smaller cochlear dimensions were associated with deeper estimated AID and increased trauma. Lower lateral scala tympani height was found for the translocations compared to non-translocations, indicating an influence on trauma risk.

**Conclusion:**

Robotic-assisted cochlear implantation resulted in less trauma occurrence compared to manual insertion. However, trauma risk was strongly influenced by cochlear geometry and insertion depth. These findings indicate that cochlear geometry is associated with insertion trauma and may support future patient-specific planning and robotic-assisted insertion strategies to reduce trauma.

**Supplementary Information:**

The online version contains supplementary material available at https://doi.org/10.1007/s00405-026-10613-1.

## Introduction

Hearing loss is the most common sensory deficit and affects over 1.5 billion people worldwide. It is associated with social isolation, cognitive impairment, and depression and can severely impact the quality of life if left untreated [1, 2]. Cochlear implants (CIs) are designed to electrically stimulate the auditory nerve and bypass normal hearing mechanisms [3]. CIs are a widely used treatment option for patients with severe to profound hearing loss and it significantly improves the quality of life for patients who do not benefit from conventional hearing aids [4, 5]. Soft surgical techniques, preserving inner ear structures and residual acoustic hearing have become a point of interest in CI studies and multiple strategies to minimize the trauma during electrode insertion have been tested and developed [5].

Typically, electrode arrays are inserted by hand. Manual insertions are affected by hand tremors and inconsistent speed [6]. Surgeon variability in insertion angle, speed, and force impacts outcomes, influencing the risk of intracochlear trauma. For patients, avoiding inner ear trauma is crucial as trauma is associated with less favorable postoperative rehabilitation results [5, 7]. Additionally, when patients have significant residual acoustic hearing, minimizing surgical trauma is essential. Preserved residual hearing generally enables acoustic hearing for lower frequencies combined with electrical stimulation which improves speech perception, especially in noisy environments [6]. Preservation of cochlear integrity reduces the risk of postoperative complications such as vertigo, facilitates the potential use of future regenerative or pharmacological therapies, and supports better long-term implant outcomes [2, 8].

In electrode insertion during CI surgery, some known factors predisposing to trauma are electrode design, insertion depth, speed and forces [9]. Previous studies have shown that softer and thinner electrode designs are likely to cause less trauma than stiffer electrode arrays [9]. Elevated insertion speeds can result in intracochlear pressure spikes during electrode insertion which can cause damage to the delicate structures of the sensory epithelium [10, 11]. Electrode translocation from scala tympani to scala vestibuli mix up the perilymph and endolymph causing disturbance to the intracochlear fluid homeostasis that leads to oxidative stress and hair cell apoptosis [9, 12]. Activation of proinflammatory or proapoptotic responses can result in fibrosis and ossification impairing hearing outcomes [12].

Comparative studies of manual vs robotic-assisted insertions indicate potential advantages of robotic systems, including lower insertion forces and reduced trauma ratings. By enforcing consistent insertion speeds and trajectories, robotic assistance may mitigate surgeon-related variability and reduce the likelihood of intracochlear trauma [5, 6]. Beyond surgical approach and technique, cochlear geometric factors also influence the outcome of electrode insertions. Anatomical and geometric parameters – such as overall cochlear size, insertion depth or round window angle have been shown to significantly influence insertion forces and the risk of intracochlear trauma [13–15].

In the present study, we compared manual and robotic insertions on intracochlear trauma, while considering cochlear geometric factors.

## Materials and methods

The study was conducted at the Microsurgery Center of Eastern Finland and the Department of Otorhinolaryngology at the Kuopio University Hospital. The study fulfilled the Helsinki Declaration and received organizational approval from Kuopio University Hospital (No. 5551894) and approval for the use of cadaveric temporal bones (9202/06.01.03.01/2013).

### Cadaveric temporal bones

Twenty post-mortem, fresh-frozen human temporal bones (TBs) were collected and defrosted prior to electrode insertion. Preoperative cone-beam computed tomography (CBCT) imaging (XFi, Planmeca Oy, Helsinki, Finland) was performed to confirm normal inner ear anatomy. TBs with vestibulocochlear malformations, cochlear fibrosis, or ossification were excluded. Preoperative CBCTs were used in both groups for planning, and in the robotic insertion group, computer-aided design (CAD) renderings were used in planning of the insertion trajectory prior to the insertion. Ten pairs of TBs with similar cochlear dimensions were selected for robotic and manual insertions. Due to exclusion criteria, pairs 3, 5 and 10 were obtained from different cadavers, whereas the remaining pairs originated from the same cadaver heads. To ensure methodological comparability, one specimen of each pair was assigned to robotic insertion and the contralateral specimen to manual insertion, resulting in five left and five right TB insertions for each group.

### Robotic system & surgical approach

The robotic-assisted insertion system iotaSOFT® (iotaMotion, Inc.) used in this study is an FDA-cleared device designed to assist in cochlear implantation. The system is mounted on the mastoid rim and inserts the electrode array at a controlled, constant speed to minimize unintended movement and surgeon tremor [5]. The device is powered and controlled by the surgeon via a foot pedal. Insertions speed is predetermined with a control console and can be adjusted any time during insertion. The drive unit base is secured to the mastoid rim with bone screws and is removed after completion of insertion. The drive unit clips mechanically into the unit base. The drive head trajectory can be manipulated manually by the surgeon to achieve the desired insertion angle. The electrode array is loaded to the drive head and positioned so that the electrode array enters the cochlea via the round window.

Mastoidectomy and posterior tympanostomy were performed under the operating microscope to gain access to the round window. All insertions were carried out using the HiFocus SlimJ electrode array (Advanced Bionics, Valencia, California, USA). The HiFocus SlimJ is a 23 mm long straight lateral wall electrode with 16 active and 1 dummy electrode contacts and has a curved tip [16]. A total of 20 insertions were performed, with ten electrode array insertions using the iotaSOFT® robotic insertion system and ten arrays inserted manually using forceps. The insertions were recorded via the operating microscope and insertion times were documented.

In the robotic-assisted group, insertions were continued until either full insertion was achieved or electrode buckling occurred. Upon observing buckling, the electrode array was carefully withdrawn by one or two contacts using the robotic system. The insertion angle was subsequently adjusted manually by adjusting the drive head, and insertion was resumed. If buckling occurred again, the insertion was terminated and the procedure concluded.

Manual insertions were performed according to structure and hearing preservation techniques, consistent with standard operating room practice. Manual insertions were carefully performed using forceps until resistance was encountered or a full insertion was achieved. If the electrode did not advance smoothly, the insertion angle was adjusted and the insertion was resumed. Following insertion, the electrode arrays were secured in place with cyanoacrylate adhesive to the posterior tympanostomy.

### Imaging & evaluation

In addition to preoperative CBCTs with a resolution of 0.3 mm (pixel spacing) × 0.3 mm (slice thickness), postoperative CBCT images with 0.075 mm × 0.075 mm resolution were acquired to confirm correct placement of the electrode. After electrode implantation a MicroCT was taken. Prior to postoperative MicroCT (XT H 225, Nikon Metrology NV, Leuven, Belgium) imaging, the perilymph was removed via the round window and a canalostomy to the lateral semicircular canal to enhance visualization of the basilar membrane. The MicroCT image resolution was set to 0.015 mm × 0.015 mm.

Cochlear A-values and B-values were taken on pre-operative CBCTs. A-values and B-values were measured after the definition of Escude et al. [17]. The A-value was measured from the round window through the modiolar axis to the lateral wall. The B-value was taken perpendicularly to the A-measure through the modiolar axis from lateral wall to lateral wall. Angular insertion depth (AID) was measured on postoperative CBTS. In addition, the number of extra-cochlear contacts were counted and compared. Trauma scoring was performed on micro-CT images using the three-tier Electrode Scalar Location (ESL) grading scale [18],where ESL 0 indicates electrode placement fully within the scala tympani (*no trauma*), ESL 1 indicates contact with the basilar membrane or osseous spiral lamina (*potential trauma*), and ESL 2 indicates translocations into the scala media or scala vestibuli (*trauma*). Each contact of an electrode array was evaluated using this scale.

### Statistical analysis

**Fig. 1:**
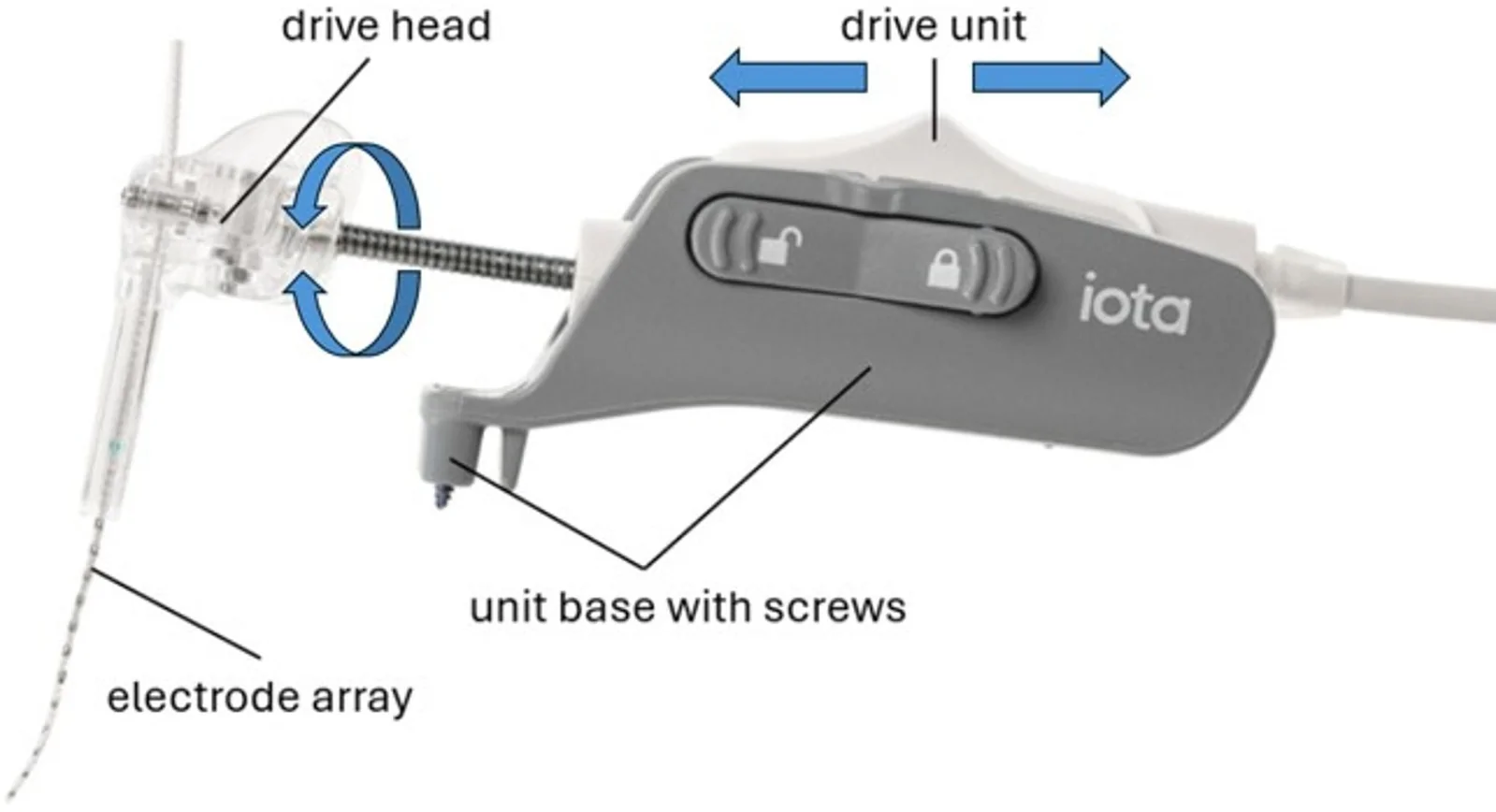
Image shows the used iotaSOFT® robotic insertion system. It consists of a unit base which is screwed onto the skull. The drive unit is clipped into the base unit and is connected to the drive head. The drive unit is actuating the drive head, which holds the electrode array, allowing the electrode array to be advanced slowly into the cochlea. The blue arrows indicate the adjustment possibilities that the surgeon can manually alter

All statistical analyses were performed using Minitab Statistical Software (Version 20). Paired comparisons were conducted using the Wilcoxon signed-rank test, while unpaired group comparisons were analyzed using Mann–Whitney tests as appropriate. Correlation analyses were performed using Pearson’s correlation coefficient. Most analyses included all insertions; however, selected analyses were restricted to full insertions to avoid bias introduced by incomplete insertions. Full insertions were defined as cases in which all 16 electrode contacts were fully located within the cochlea (Fig. 1).

### Imaging tool

**Fig. 2:**
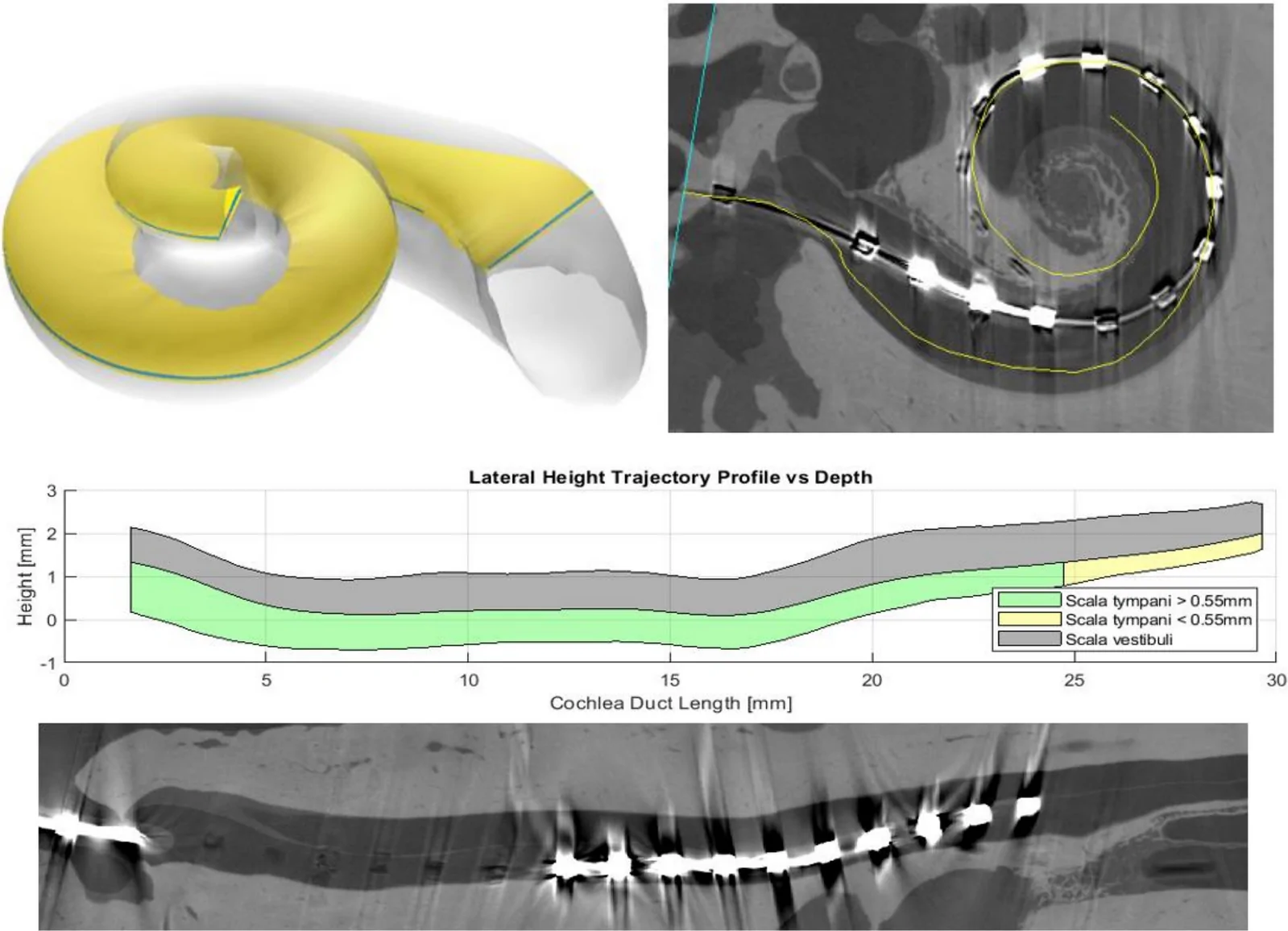
The top left image shows the generated cochlea model from the imaging tool. The image on the top right is the postoperative micro-CT image. The two bottom figures display the cochlea in an unrolled representation, while one of them is from the imaging tool, the other from the micro-CT image. The cochlea is cut along the lateral wall (yellow line in top right image). The scala vestibuli is represented in grey, the scala tympani is shown in green and yellow. If the lateral height is above 0.55 mm, which is the largest dimensions of the electrode tip, the scala tympani is shown in green. If the scala tympani lateral height is lower than the electrode tip dimensions, the scala is represented in yellow as potential basilar membrane lifting or rupture might appear. Below is a cut along the lateral wall shown from the Micro-CT. The basilar membrane can be seen as a thin line. The cut does contain the electrode array

A cochlear implant imaging tool developed by Advanced Bionics (Valencia, CA, USA) enables the generation of patient-specific cochlea models from preoperative CT scans, as well as electrode array models from postoperative CT scans [18–20]. The tool generates an individual cochlea model and predicts the locations of the basilar membrane and osseous spiral lamina. Based on the extracted cochlear geometry, the tool estimates the angular insertion depth (AID) for each individual cochlea assuming full electrode insertion. In addition, it provides quantitative information about the scala tympani height along the lateral length, as illustrated in Fig. 2. This information can be used to assess whether the scala tympani provides sufficient space to accommodate a given electrode array. The HiFocus SlimJ electrode has tip dimensions of 0.26 × 0.55 mm. When the lateral ST height decreases below 0.55 mm, the electrode tip may no longer fit entirely into the scala tympani, increasing the risk of basilar membrane elevation or rupture or even translocations. Within the imaging tool, regions of the scala tympani with a lateral height exceeding 0.55 mm are displayed as green, whereas regions below this threshold are displayed in yellow (Fig. 2).

Given the substantial inter-individual variability in cochlear anatomy, this tool was used to compare cochlear geometry between robotic-assisted and manual insertion groups and to analyze the influence of anatomical factors on trauma occurrence. Specifically, the estimated angular insertion depth, the lateral scala tympani height along the cochlear length, and the angular position at which the lateral height fell below 0.55 mm (0.55 mm marker) were evaluated.

## Results

All 20 electrode insertions were performed via the round window approach. Full insertion was achieved in 9 of the 20 TBs, with incomplete insertions occurring more frequently in the robotic-assisted group (8 robotic vs. 3 manual). One specimen (cochlea 5 M) ex habited a malformation of the cochlear structures and was therefore excluded from statistical analysis. No electrode tip-fold-overs were observed in either group.

**Fig. 3:**
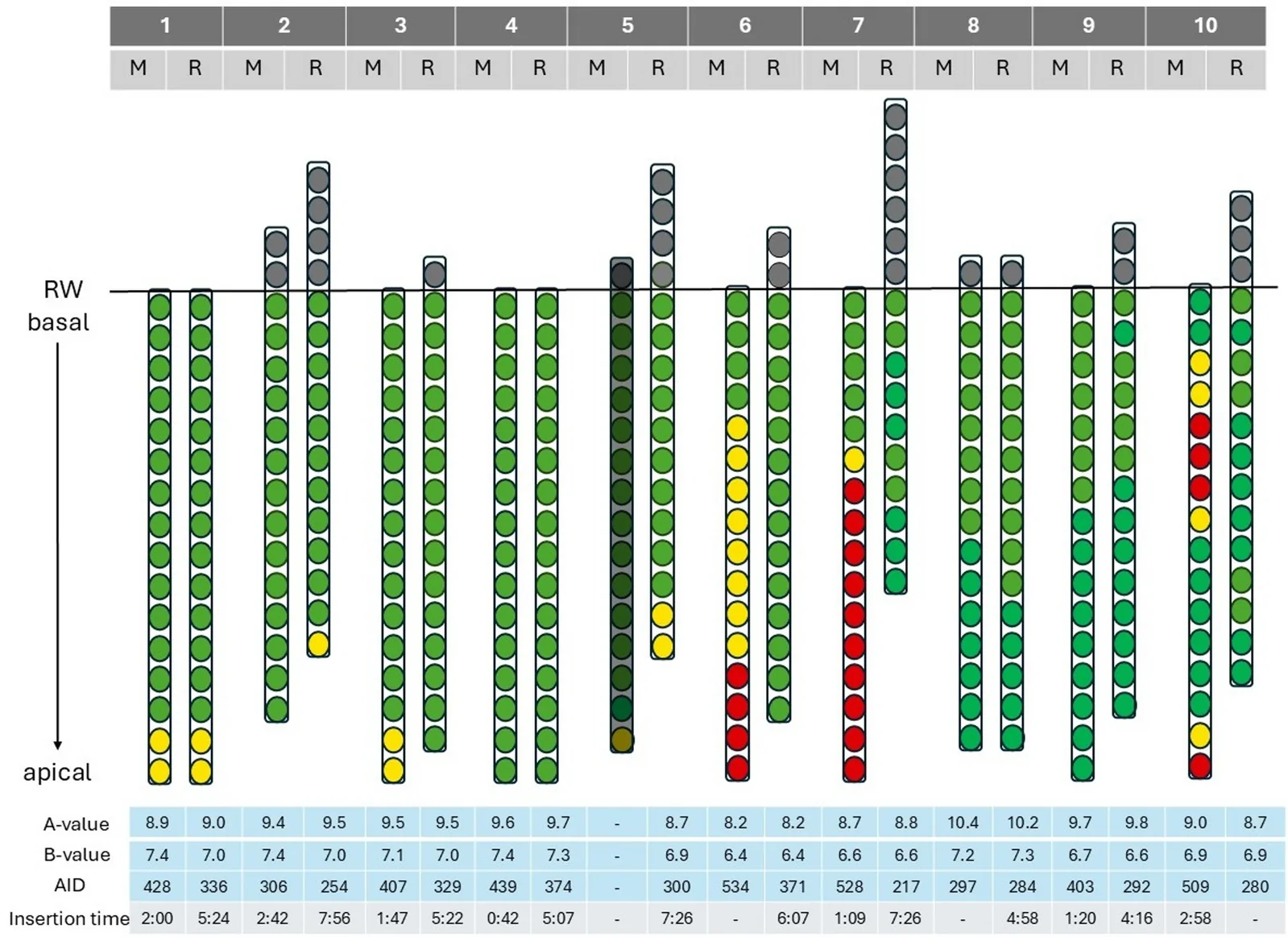
Figure shows the ten groups of cochlea pairs with one cochlea inserted manually (M) and one with robotic assistance (R). Each dot represents one of the 16 electrode contacts of the HiFocus SlimJ electrode. The color indicates the trauma scoring (ESL 0 = green, ESL 1 = yellow, ESL 2 = red). Grey dots represent contacts outside of the round window (RW). For each cochlea the A-value, B-value, angular insertion depth (AID) and insertion time are shown in the table

The trauma assessment based on micro-CT images revealed no scalar translocations in the robotic insertion group. In contrast, three translocations into the scala media or scala vestibuli were identified in the manual insertion group. The mean electrode scalar location (ESL) score was 0.30 ± 0.48 for the robotic group and 0.89 ± 0.93 for the manual group. Trauma outcomes, A-values and B-values, angular insertion depth (AID) and insertion times are summarized in Fig. 3. Electrode scalar location is depicted using color-coded markers (ESL 0 = green, ESL 1 = yellow, ESL 2 = red).

Mean A-value was 9.3 ± 0.6 mm for the manual group and 9.2 ± 0.6 mm for the robotic group, while B-values were 7.0 ± 0.4 mm and 6.9 ± 0.3 mm, respectively. There was no significant difference between the groups in the A-values (p = 0.935) nor the B-values (p = 0.410). The mean angular insertion depth (AID) was 428 ± 82 degrees for manual and 304 ± 47 degrees for the robotic-assisted insertion group. The difference in AID between groups was significant (p = 0.004). The robotic-assisted group had significantly (p = 0.011) more extra-cochlear contacts with a mean of 2.3 ± 1.8 compared to the manual group with 0.3 ± 0.7.

A strong positive correlation was observed between AID and trauma scores (r = 0.682, p = 0.001). The A-value demonstrated a moderate negative correlation with trauma scores (r = −0.494, p = 0.032), whereas the B-value did not demonstrate significant correlation with trauma scores (r = −0.278, p = 0.249). The number of extra-cochlear contacts was strongly negatively correlated with AID (r = −0.747, p < 0.001).

When the analysis was restricted to full insertions to account for potential bias introduced by incomplete insertions, the strength of these relationships increased. AID remained strongly positively correlated with trauma (r = 0.733, p = 0.025), while both cochlear geometric measures showed strong negative correlations with trauma, with r = −0.865 (p = 0.003) for the A-value, while the correlation for the B-values was not significant (r = −0.602, p = 0.086).

Insertion time was measured from the moment the electrode was positioned at the round window until removal of the forceps from the electrode. The time could not be obtained from two manual and one robotic-assisted insertion videos due to technical difficulties or file corruption. There was a significant difference in insertion time between the robotic group with a mean of 351 ± 85 s and manual with a mean of 108 ± 46 s (p = 0.001). Despite this difference, no correlation was observed between insertion time and trauma scores when considering all insertions (r = −0.151, p = 0.576). Similarly, no correlation was found when the analysis was restricted to full insertions (r = −0.045, p = 0.015).

### Imaging tool

Using the imaging tool, preoperative CT scans were used to generate patient-specific cochlea models for the analysis of geometric factors. One parameter derived from these models is the lateral scala tympani height along the arc length of the cochlea. The HiFocus SlimJ electrode has a width of 0.55 mm at the tip and 23 mm length.

**Fig. 4:**
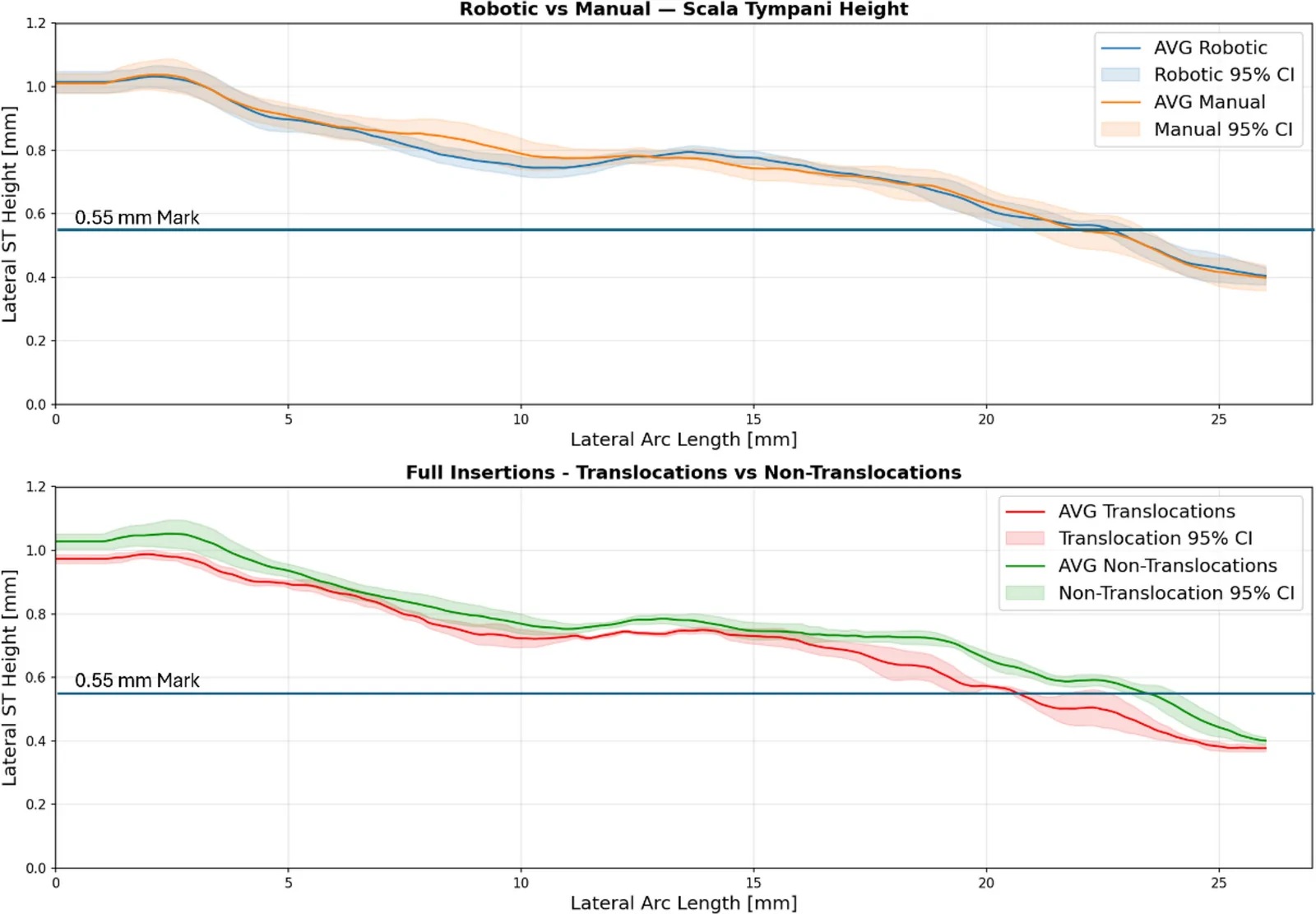
The top diagram shows the average and 95% confidence interval of the lateral scala tympani height for the robotic and manual group. The intervals are overlapping, indicating no relevant difference in ST height. The bottom diagram shows the average and 95% confidence interval of the lateral scala tympani height between translocation and non-translocation cases for full insertions. The graphs show no overlapping in deeper regions of the cochlea duct, indicating lower ST height for the trauma cases. Additionally, the 0.55 mm mark is added, which represents the dimensions of the electrode tip

Figure 4 illustrates the lateral scala tympani height over the cochlear arc length. Comparisons were performed between the robotic and manual cases, as well as between the translocation and non-translocation cases restricted to full insertions. The robotic and manual groups demonstrated similar lateral ST height profiles, with overlapping 95% confidence intervals along the cochlear length, indicating no relevant anatomical differences between the groups. In contrast, translocation and non-translocating cases showed marked differences, with largely non-overlapping 95% confidence intervals across most regions of the cochlea. The translocation cases had a consistently lower scala tympani height along the lateral arc length compared with non-translocation cases.

Estimated AID did not differ significantly between robotic-assisted and manual insertion groups (paired: p = 0.953). A negative correlation was observed between cochlear A-value and estimated AID (r = −0.914, p = < 0.001), as well as between B-value and estimated AID (r = −0.712, p = 0.001). No significant difference was found between measured AID and estimated AID for manual insertions (p = 1.00). In contrast, for the robotic group the estimated AID was significantly higher than the measured AID (p = 0.006).

Positive correlations were identified between cochlear size and the angular position of the 0.55 mm marker, with r = 0.608 (p = 0.006) for A-value and r = 0.722 (p < 0.001) for B-value. Furthermore, the difference between the measured AID and the 0.55 mm marker demonstrated a negative correlation with trauma scores (r = −0.683, p = 0.001), indicating that trauma severity increased as the measured AID approaches or exceeded the 0.55 mm marker. Notably, in all observed translocations (manual insertions 6,7, and 10), the measured AID exceeded the angular position of the 0.55 mm marker. The values are summarized in Table 1.

**Table 1:** The table does compare measured outcomes with pre-operative imaging data. Shown are measured AID and estimated AID if fully inserted. The robotic cases had a mean of 131 degrees difference to the estimated AID, while the manual cases had a mean difference of 9 degrees. Additionally, the imaging tool gives an angle of degree when the required 0.55 mm ST height is not given for the specific cochlea anymore. For the three translocations cases, the 0.55 mm Mark was exceeded while for none of the non-translocating cases the mark was exceeded

	Robotic assisted			Manual			
	*Estimated AID [°]*	*Measured AID [°]*	*0.55 mm* *Mark [°]*	*Estimated AID [°]*	*Measured* *AID [°]*		*0.55 mm Mark [°]*
*No*							
1	425	336	450	423	428		448
2	433	254	442	438	306		452
3	416	329	452	425	407		454
4	398	374	452	411	439		452
5	461	300	426	Malformation			
6	497	371	378	527*	534	342	
7	489	217	380	456*	528	442	
8	367	284	462	363	297	442	
9	416	292	436	413	403	438	
10	447	280	466	434*	509	444	
MEAN	435	304	434	432	428	434	
SD	38	47	30	41	82	35	
				*Translocation			

## Discussion

Robotic-assisted insertion systems have been developed to reduce insertion forces and variability compared to conventional manual techniques [7]. Previous studies have evaluated the difference between robotic and manual insertions on 3D-printed inner ears, cadaveric temporal bones, and clinical trials [5–7]. The objective of the present study was to compare insertion results of manual and robotic-assisted insertions in human temporal bones, while considering cochlear geometric factors.

When looking at the trauma, we could find three cases of electrode array translocations in the manual insertion group, whereas no translocations occurred in the robotic group. Previous investigations by Kaufmann et al. [5] and Claussen et al. [6] similarly compared manual and robotic insertions, using cadaveric temporal bones and whole cadaver heads, respectively. However, both studies employed a different trauma grading scale, which limits direct comparison to our findings. Nevertheless, Kaufmann et al. and Claussen et al. reported significant higher trauma scores overall for the manual insertions, indicating more severe trauma. While statistical testing was not emphasized, the distribution of trauma grades and means suggests a trend towards a reduced trauma for robotic insertions. Daoudi et al. [21] investigated a different robotic-assisted insertion platform. Using the HiFocus SlimJ, they observed one translocation in eight robotic-assisted insertions, compared with three translocations in sixteen manual insertions.

Kaufmann et al. [5] did not observe a statistically significant difference in AID between manual and robotic insertions, whereas in the present study a significantly greater AID was achieved for the manual insertions compared to robotic insertion. Similar to our methodology, Kaufmann et al. terminated insertions upon detection of increased resistance or electrode buckling. Claussen et al. [6] reported similar AID for both groups but do not provide statistical testing and noted difficulty in angle measurement in one case. Moreover, Claussen et al. did not specify an insertion stop criteria, limiting the interpretability of their findings.

The mean AID achieved in our manual insertion group (428 ± 82°) is higher than that reported for manual insertion in previous studies, including 359 ± 113° in Kaufmann et al. [5] and 311 ± 131° Claussen et al. [6]. Conversely, the mean AID in our robotic group (304 ± 47°) is comparable to values reported by Kaufmann et al. (321 ± 84°) and Claussen et al. (307 ± 96°). Daoudi et al. [21] did find an average AID of 440 ± 28.2° for the robotic group and 425 ± 11.1° for the manual group. In our study, a strong positive correlation between AID and trauma scores was observed. This finding raises the question of whether the increased trauma for the manual group is primarily attributable to the greater insertion depth rather than the insertion technique itself. Notably, neither Kaufmann et al., Daoudi et al. nor Claussen et al. reported analyses correlating AID with intracochlear trauma.

Another notable finding was the significant difference in the number of extra-cochlear contacts between the manual and robotic groups. The robotic group exhibited a mean of 2.3 ± 1.8 contacts outside the cochlea, compared with only 0.3 ± 0.7 contacts in the manual group. From a clinical perspective full electrode insertion is generally desirable, as extra-cochlear contacts have been associated with poorer performance. [22, 23] However, in the present study, suspected insertion trauma manifested as electrode buckling and frequently resulted in incomplete insertion, thereby increasing the number of extra-cochlear contacts in the robotic group. Although this scenario does not fully reflect routine clinical practice, it highlights a potential advantage of robotic insertion systems: the ability to detect insertion-related events that may indicate an increased risk of intracochlear trauma. The identification of such events remains clinically relevant even when accompanied by incomplete insertion, particularly in electroacoustic stimulation (EAS) candidates undergoing planned partial insertions, where the preservation of residual hearing is crucial. [24, 25]

Kaufmann et al. [5] report that robotic insertions performed at lower insertion speeds lead to significantly reduced insertion forces compared with manual insertions. Higher insertion forces have been associated with increased risk of intracochlear trauma, leading the authors to the conclusion that increased insertion forces may contribute to higher trauma occurrence. However, their study does not show if there is a direct correlation between insertion speed and trauma outcomes. In the present study, insertion speed and insertion forces were not directly assessed. Instead, the time of insertion for each group was evaluated, as this parameter more closely reflects the clinical workflow. No correlation between the insertion time and trauma was observed.

In contrast to previous studies, we accounted for cochlear geometric factors in our analysis. Specimens were selected to form matching pairs between robotic assisted and manual insertion groups. The absence of statistical differences in A-values, B-values and the estimated AID between the groups indicates successful matching was achieved with respect to these anatomical parameters. In addition to comparing robotic and manual insertions, we performed a separate analysis restricted to full insertions as translocations usually appear in apical regions and incomplete insertions would impact the statistical comparison.. It should be noted that the subgroup of full insertions was considerably smaller than the overall robotic and manual cohort, comprising only nine in total, including three translocations and six non-translocations. Consequently, the statistical power of analyses performed within this subgroup is limited, and the results should therefore be interpreted with caution.

When considering all insertions, correlations between cochlea size and trauma were moderate to weak, with A-values showing moderate negative and B-values a weak negative correlation with trauma scores. However, when the analysis was limited to full insertions, both A- and B- values demonstrated strong negative correlation with trauma. Furthermore, both parameters exhibit strong negative correlation to the estimated AID, indicating that smaller cochleae are associated with deeper insertions and, consequently, an increased risk of trauma as previously stated. Although both geometric measures are strongly associated with trauma and estimated AID, the A-value exhibits a particularly high correlation, suggesting that the A-value plays a dominant role in influencing insertion depth and trauma risk.

Another anatomical factor examined is the lateral scala tympani height, which corresponds to the region occupied by the electrode array within the cochlea. Progressive narrowing of the lateral ST reduces the available intracochlear space for the electrode array and may increase the chance of basilar membrane elevation or translocation. Comparing the lateral ST height for the robotic group and manual group demonstrates comparable ST height along the cochlear length. Comparison of the ST height between the translocation cases and the non-translocation cases in the full insertions group demonstrates consistently lower lateral ST heights across most cochlear regions.

Attention was given to the 0.55 mm marker, corresponding to the tip width of the HiFocus SlimJ electrode. This marker represents the cochlear depth beyond which the electrode theoretically no longer has sufficient space to remain fully withing the scala tympani, increasing the risk of ST elevation or translocations. All three observed translocation cases exhibit measured AID exceeding the 0.55 marker, whereas non-translocation cases remained below the threshold. Furthermore, a strong negative correlation was identified between the difference of measured AID relative to the 0.55 mm marker and trauma scores. This finding suggests that as the electrode approaches or surpasses the depth at which the available ST height becomes insufficient, the likelihood and severity of intracochlear trauma increase. Strong positive correlations were observed between both A- and B-values and the 0.55 mm marker, indicating that larger cochleae are generally associated with greater ST height. Notably, the B-value shows a slightly stronger correlation with this marker than the A-value.

Overall, lower trauma scores were observed in the robotic-assisted group compared with the manual insertion group. However, based on the present data, it cannot be conclusively determined whether this difference or attributable to the robotic insertion technique itself or to underlying cochlear geometric factors that predispose to increased trauma. Our analyses indicate that greater angular insertion depth and reduced lateral ST height are both associated with a higher likelihood of intracochlear trauma. Longer insertion times seen in robotic insertions on the other hand did not correlate with trauma.

The findings underscore the importance of incorporating individual cochlear geometry into insertion planning to minimize intracochlear trauma. The integration of patient-specific imaging with controlled robotic-assisted insertion strategies may present a promising approach for optimizing electrode placement while reducing trauma risk. Such advancements have the potential to increase surgical outcomes and ultimately improve postoperative hearing preservation and hearing improvement.

A strength of this study is the matched temporal bone specimens. This matching decreased the variability between robotic-assisted and manual groups. Furthermore, we incorporated individual cochlear geometric factors in our data, allowing a more detailed interpretation of insertion outcomes and trauma risk. However, the study was limited by its relatively small sample size, limiting the robustness of statistical analyses. Therefore, additional data – particularly from in vivo studies that also account for patient-specific cochlear geometry – are required to further analyze the impact of robotic-assisted insertion on surgical outcomes and intracochlear trauma.

## Conclusions

Robotic-assisted cochlear implant insertions resulted in lower trauma scores compared with manual insertions in human temporal bones, although with a higher rate of incomplete insertions. While it remains unclear whether the reduction is a result of the robotic technique itself or to cochlear geometric factors, greater angular insertion depth and reduced lateral scala tympani height were strongly associated with increased trauma. These findings demonstrate that cochlear geometric factors are associated with insertion trauma. They provide a foundation for future studies evaluating whether patient-specific planning combined with robotic-assisted insertion techniques can help to minimize intracochlear trauma.

## Supplementary Information

Below is the link to the electronic supplementary material.

Supplementary Material 1 (MP4 257 MB)
